# Supplementary material for: Oxidation-boosted charge trapping in ultra-sensitive van der Waals materials for artificial synaptic features
Source: Nat Commun. 2020 Jun 12;11:2972. doi: 10.1038/s41467-020-16766-9 (PMC7293344; doi:10.1038/s41467-020-16766-9)
Supplement: Supplementary file 1 — Supporting Information [file 41467_2020_16766_MOESM1_ESM.pdf]

Supplementary Information

Oxidation-boosted charge trapping in ultra-sensitive van der Waals materials for artificial synaptic features

Yang *et al*.

1. Supplementary Figures

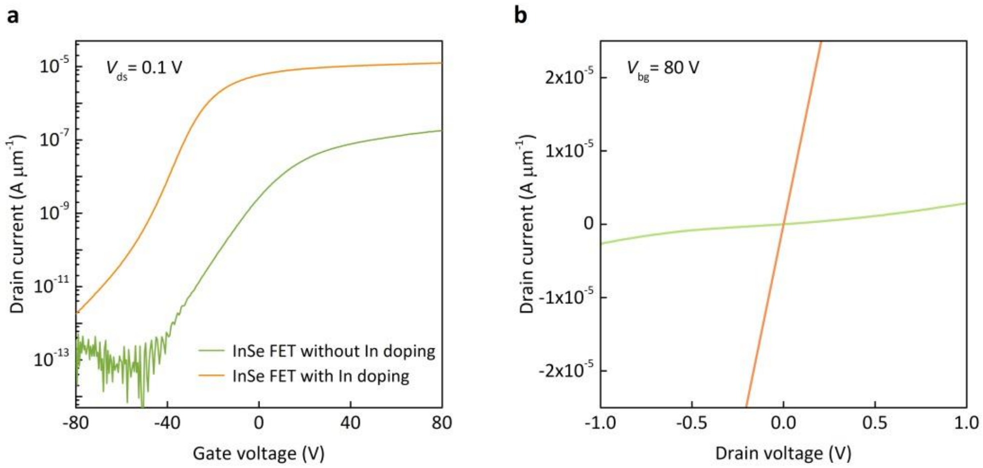

Supplementary Figure 1. Electrical curves for InSe FETs under vacuum conditions. a, Transfer characteristics for two devices with/ without In doping layer. The value of drain current for the InSe FET is largely increased by introducing 32-nm-thick In surface doping layer. **b**, Corresponding output curves for two devices with/ without In doping layer, which indicates the better contact behaviour between source/ drain electrodes and InSe channel by depositing the In doping layer.

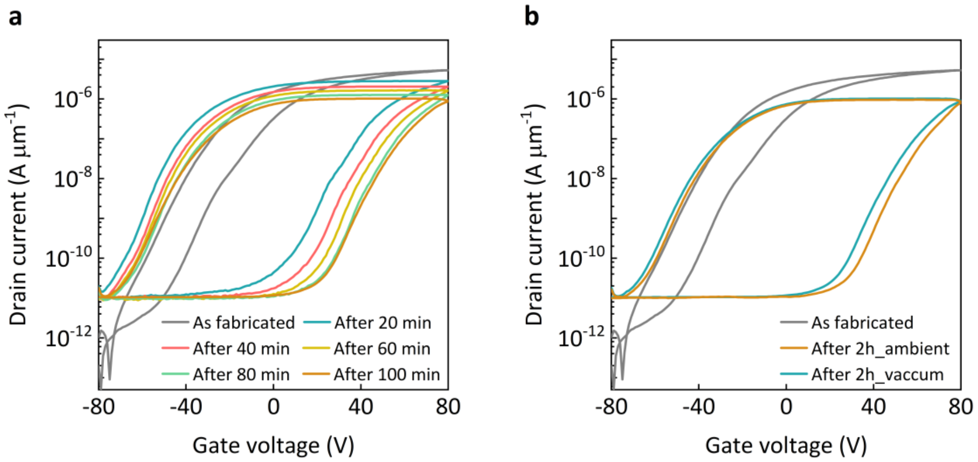

Supplementary Figure 2. The measured transfer curves depending on various conditions. **a,** Transfer characteristics for InSe FET at different exposing-time conditions. Note that all of the curves were measured under vacuum conditions after immersing in ambient conditions for various exposing times to minimize the occurrence of oxidation during the electrical measurement process in air. **b,** The transfer curves recorded under ambient conditions (Brown) and vacuum conditions (Turquoise) after immersing in ambient conditions for 2 hours, respectively. Note that the drain-source voltage is set as 0.1 V.

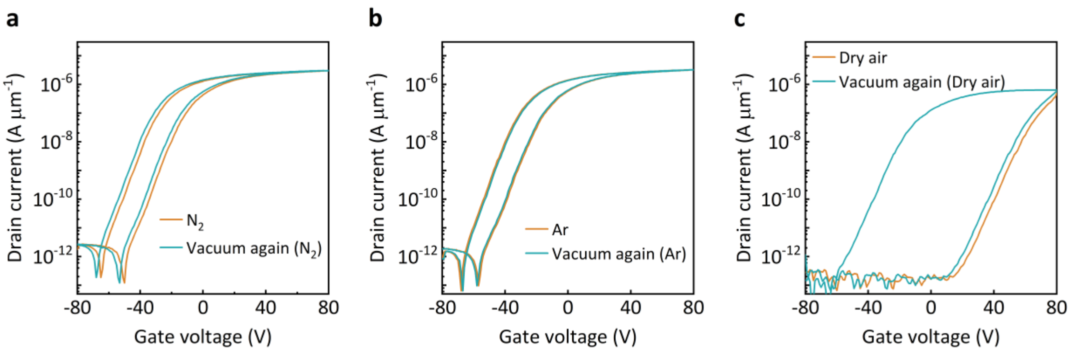

Supplementary Figure 3. Transfer characteristics of InSe FET under various conditions. a, Transfer characteristics under N_2 for around 30 min and stored back to vacuum again. **b,** Transfer characteristics under Ar for around 30 min and stored back to vacuum again. **c,** Transfer characteristics under dry air (30% O_2 and 70% N_2) for around 30 min and stored back to vacuum again. The above results indicate that the enhanced hysteretic features are contributed from the native oxide layer, instead of the physical absorption. Note that the drain-source voltage is set as 0.1 V.

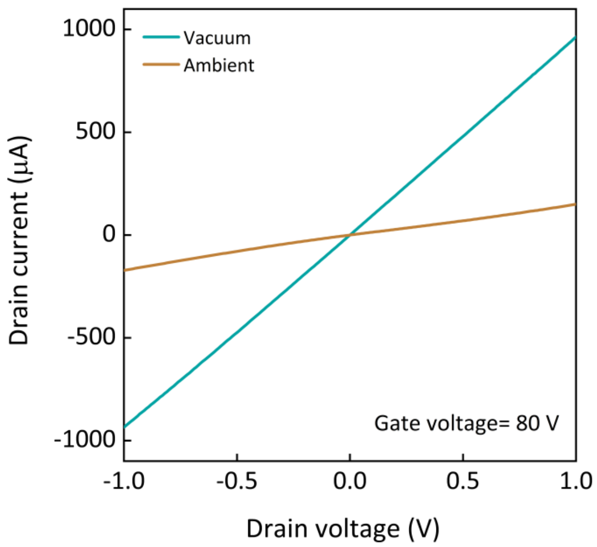

Supplementary Figure 4. Output characteristics of InSe FET under different conditions. The output curves for InSe V- and A-FET, respectively.

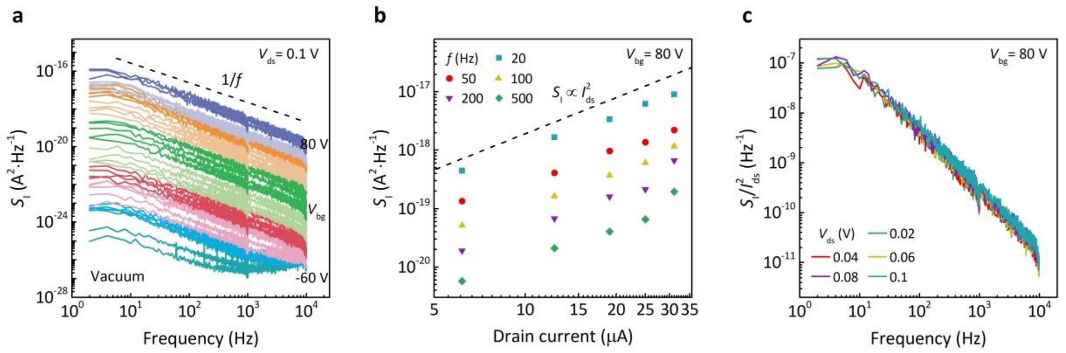

Supplementary Figure 5. Results of dynamic characteristic analysis for InSe V-FET. a, Noise power spectra (S_I) of current fluctuations depending on the frequency of InSe V-FET under various applied V_{bg}. Note that the dashed line shows the ideal 1/*f* trend as a guide for the eyes. **b,** S_I as a function of drain current at a fixed gate voltage and various frequencies. The dashed line indicates that S_I is proportional to I_{ds}^2. **c,** Normalized S_I versus frequency for InSe V-FET at different drain voltage values. As we can see, all of the normalized curves appear to overlap together, which indicates the better contact behaviour at the InSe channel and metal electrodes in InSe V-FET.

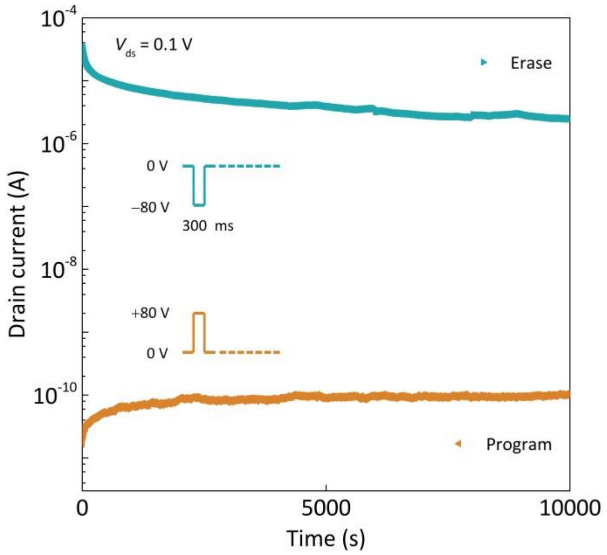

Supplementary Figure 6. Retention characteristics of InSe A-FET based memory. The read currents were recorded at V_{bg}= 0 V and V_{ds}= 0.1 V.

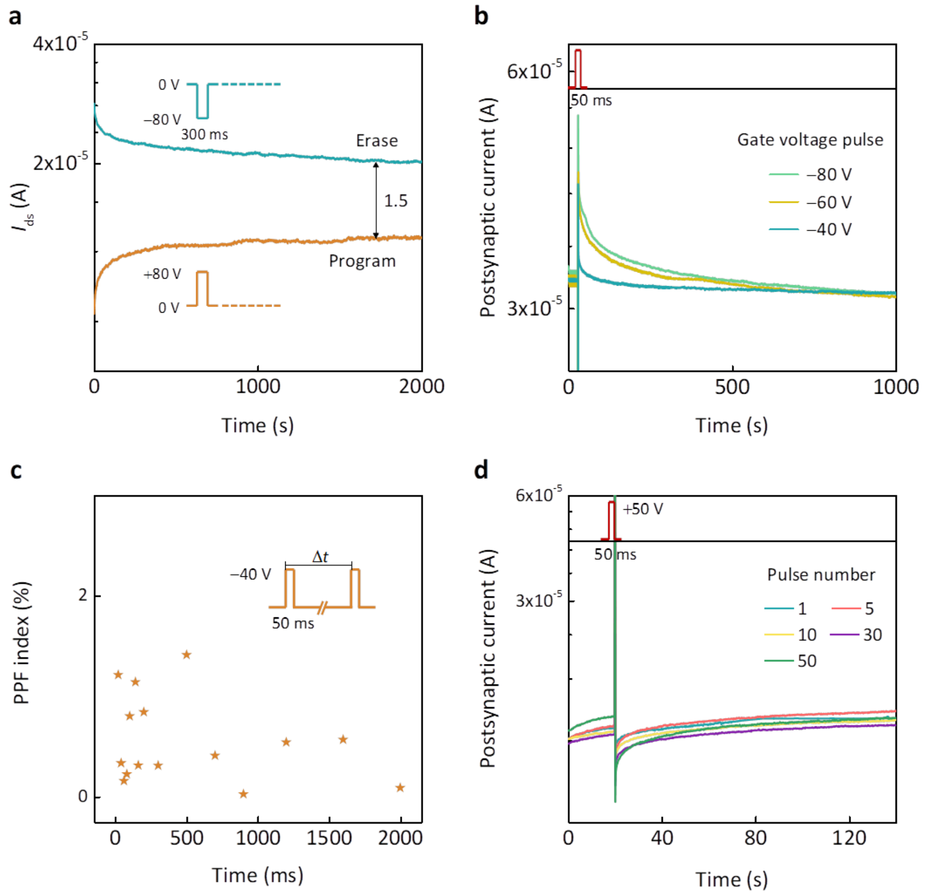

Supplementary Figure 7. The related demonstration based on InSe V-FET device. a, The retention ability of InSe V-FET based memory device. The read currents were recorded at V_{bg}= 0 V and V_{ds}= 0.1 V. **b,** The postsynaptic currents generated by the application of input spikes (−40 V to −80 V). Note that the drain voltage is set as 0.1 V under the Read operations. **c,** The extracted values of PPF index depending on the increasing time interval, which show slight variations near 0. **d,** The generated postsynaptic currents under sequential simulation with various pulse numbers.

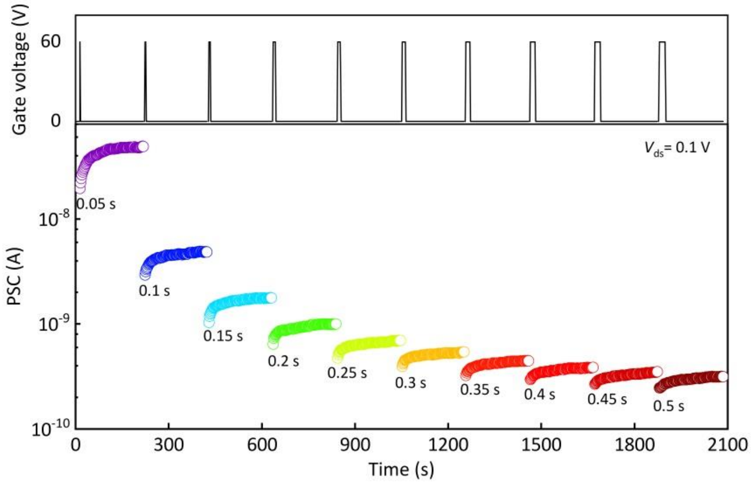

Supplementary Figure 8. Evolution of IPSC responses under different pulse widths. The recorded IPSC responses of the InSe artificial synaptic device depending on reading time with different input widths, which indicate the controllable memory characteristics of the conductance of the InSe channel.

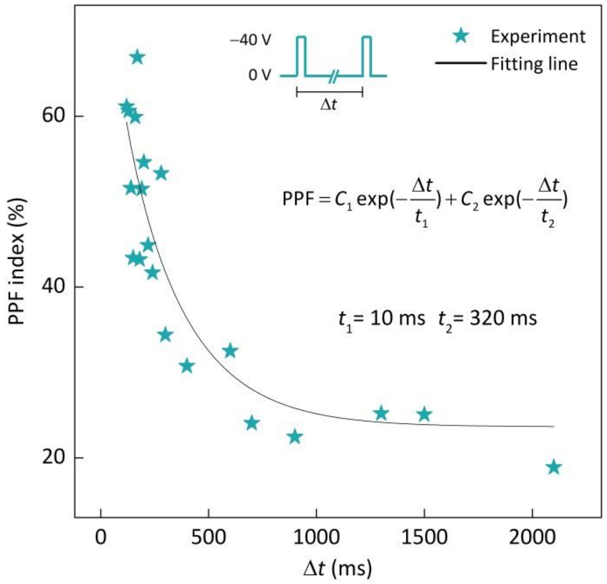

Supplementary Figure 9. Calculated PPF index as a function of time interval. The fitted result of PPF index is presented by the solid line according to the double exponential function of $\mathrm{PPF} = C_1 \exp(-\frac{\Delta t}{t_1}) + C_2 \exp(-\frac{\Delta t}{t_2})$, where C_1 and C_2 are two facilitation parameters, and t_1 and t_2 are two relaxation time constants, respectively. As a result, the relaxation time constants can be extracted as t_1= 10 ms and t_2= 320 ms, which are similar to those of a biological synapse.

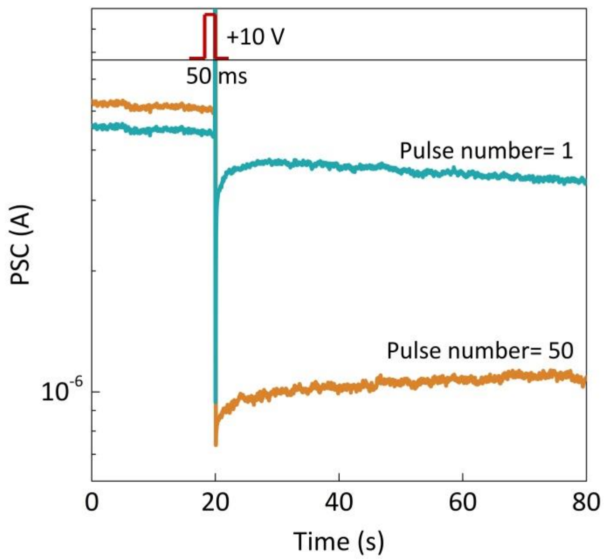

Supplementary Figure 10. IPSC responses under different input pulses. The plot of IPSC changes as a function of retention time over 80 s after simulation using a certain number of input spikes (Pulse number= 1 and 50). It can be observed that when pulse number= 1, the recorded IPSC sharply decreases and then climbs back quickly to the initial states, which indicates the emulation of the STP behaviour. While after suitable training (pulse number= 50), the recorded IPSC undergoes a sharp decrease and can be well-maintained over the measurement periods, which suggests the emulation of the LTP behaviour.

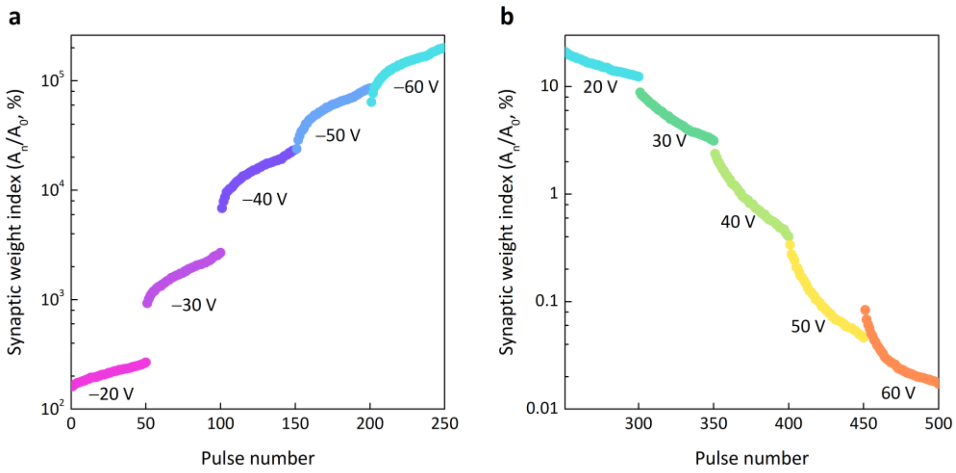

Supplementary Figure 11. The evolution of the synaptic weight under successive stimuli. a, The evolution of the calculated synaptic weight (A_n/A_0) depending on the number of successive input pulses. Note that the input gate voltages are varied from −20 to −60 V. **b,** The corresponding synaptic weight under the stimulation of successive positive voltage pulses varying from 20 to 60 V.

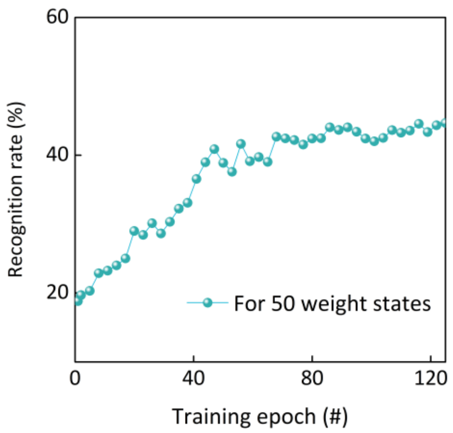

Supplementary Figure 12. Recognition rates for 50 weight states. The obtained recognition rates as the function of training phases by testing 10000 images for 50 weight states.

2. Supplementary Notes

Supplementary Note 1. The related dynamic characteristic measurements based on low-frequency noise (LFN) technology:

LFN is considered as a powerful tool to diagnose the dominant mechanisms of carrier fluctuations in the active regions of electronic devices, in particular, for the layered conducting channels because of the enhanced surface states. In conventional metal-oxide-semiconductor field-effect transistors (MOSFETs), the carrier number fluctuation is derived from the dynamic carrier trapping and releasing events near the interface between the dielectric and semiconductor. Furthermore, the fluctuation of the inversion layer mobility needs to be considered in terms of the fluctuations of the trap charges, which is known as the surface effect[1-5]. Regarding the unique configurations of the fabricated devices with native oxide and/or protection layer, in this work, the 1/*f* noise in these electronics was analyzed by combining the carrier number fluctuation and correlated mobility fluctuation. Thus the current noise power spectral density (S_I) normalized by drain current (I_{ds}) is given as:

$$S_I I_{ds}^{-2} = (1 + \alpha_{sc}\mu_{eff}C_{ox}I_{ds}g_m^{-1})(g_m I_{ds}^{-1})^2 S_{Vfb} \quad \text{(Supplementary Equation 1)}$$

$$S_{Vfb} = q^2 k_B T N_{it}(fWLC_{ox}^2)^{-1} \quad \text{(Supplementary Equation 2)}$$

where α_{sc}, μ_{eff}, C_{ox}, g_m are the mobility fluctuation associated Coulomb scattering coefficient, the effective mobility, the gate capacitance per unit area, and the gate transconductance, respectively. S_{Vfb} is the flat-band voltage spectral density, where q, k_BT, N_{it}, f, W, and L are the elementary charge, the thermal voltage, effective trap density, the frequency, the channel width, and the length, respectively. The governing mechanism of the 1/*f* noise signals in electronic devices can be interpreted by plotting the log-log scaled S_I/I_{ds}^2 depending on I_{ds}. If the normalized S_I obeys the variation of $1/I_{ds}^2$, the dominant carrier fluctuation is assigned as the surface effect. It can be regarded as the bulk effect (Hooge mobility fluctuation) when the normalized S_I depends on the variation of $1/I_{ds}$.

3. Supplementary References

1. Ghibaudo, G., Roux, O., Nguyen-Duc, Ch., Balestra, F. & Brini, J. Improved analysis of low frequency noise in field-effect MOS transistor. *Phys. stat. sol.(a)* **124**, 571 (1991).

2. Sharma, D. et al. Electrical transport and low-frequency noise in chemical vapor deposited single-layer MoS_2 devices. *Nanotechnology* **25**, 155702 (2014).

3. Jang, D. et al. Low-frequency noise in strained SiGe core-shell nanowire p-channel field effect transistors. **97**, 073505 (2010).

4. Wu, W. et al. Mobility fluctuation-induced low-frequency noise in ultrascaled Ge nanowire nMOSFETs with near-ballistic transport. *IEEE Trans. Electron Devices* **65**, 2573 (2018).

5. Kwon, H.-J., Kang, H., Jang, J., Kim, S. & Grigoropoulos, G. P. Analysis of flicker noise in two-dimensional multilayer MoS_2 transistors. *Appl. Phys. Lett.* **104**, 083110 (2014).
